# Reproductive and Female Health in the Australian Defence Force 2002-2023: A Systematic Review and Synthesis

**DOI:** 10.1093/milmed/usaf411

**Published:** 2025-08-11

**Authors:** Natasha Kinsman, Helen Kelsall, Dunya Tomic, Alex Collie, Zoe Jenkins, Jace Drain, Julian Saboisky, Karen Walker-Bone

**Affiliations:** Monash Centre for Occupational and Environmental Health, School of Public Health and Preventive Medicine, Monash University, 553 St Kilda Road, Melbourne, Victoria, 3004, Australia; Monash Centre for Occupational and Environmental Health, School of Public Health and Preventive Medicine, Monash University, 553 St Kilda Road, Melbourne, Victoria, 3004, Australia; Monash Centre for Occupational and Environmental Health, School of Public Health and Preventive Medicine, Monash University, 553 St Kilda Road, Melbourne, Victoria, 3004, Australia; Healthy Working Lives Research Group, School of Public Health and Preventive Medicine, Monash University, 553 St Kilda Road, Melbourne, Victoria, 3004, Australia; Directorate of Health Research, Joint Health Command, Department of Defence, Canberra, Australian Capital Territory, 2609, Australia; Directorate of Health Research, Joint Health Command, Department of Defence, Canberra, Australian Capital Territory, 2609, Australia; Directorate of Health Research, Joint Health Command, Department of Defence, Canberra, Australian Capital Territory, 2609, Australia; Monash Centre for Occupational and Environmental Health, School of Public Health and Preventive Medicine, Monash University, 553 St Kilda Road, Melbourne, Victoria, 3004, Australia

## Abstract

**Introduction:**

Military service involves potential exposure to a range of physical, chemical, and biological hazards that could impact reproductive health. Females represent 20% of Australian Defence Force (ADF) members and, like many military services, the ADF is prioritizing their recruitment and retention. However, given that females have different physiology and anthropometry, specific female occupational research is essential. This systematic review aimed to retrieve and summarize the findings of research involving currently serving ADF personnel with outcomes relevant to male or female reproductive health or any aspect of female health.

**Materials and Methods:**

Reproductive and female health studies between January 2002 and April 2023 that included serving ADF members were identified through a systematic search of 5 scientific databases. Data were extracted and synthesized, alongside a risk of bias assessment following PRISMA-ScR guidelines.

**Results:**

Overall, 8 studies relating to reproductive health and 14 studies related to female health were identified. Studies were methodologically heterogeneous: few studies were at low risk of bias and few covered the same health outcome, preventing pooling of data. Four studies of males concluded that deployment to the Middle East was not associated with risk of infertility or birth defects. Female reproductive health studies provided very limited data. Other female health research primarily covered musculoskeletal disorders and injuries from basic training.

**Conclusions:**

Studies of reproductive and female health in ADF members do not currently provide a cohesive or comprehensive body of evidence in either area. This review serves as a systematic map of the existing evidence to identify gaps and set future strategic research agendas. More high-quality longitudinal studies with sex-stratified analyses are urgently needed, as is a strategic focus on health outcomes that may affect military preparedness and fitness for deployment.

## BACKGROUND

Although females have undertaken military roles throughout organized warfare, particularly during crises, they have historically not participated in front-line combat roles. The Australian Defence Force (ADF) began increasing integration of females in the 1970s, enabling females access to noncombat ships, but accelerated integration since the 1990s. Serving females are eligible to apply for all roles, including special forces, since 2014 and can directly enter any combat role since 2016. Currently, females represent 20.4% of ADF personnel, increased from 14% in 2012.^1^

Globally, as numbers of females in military roles increased, specific health challenges have been highlighted, some of which can be exacerbated after transition out of the forces. For example, serving female personnel may experience an increased risk of hysterectomy, reproductive ill-health and sexually transmitted infections, post-traumatic stress disorder (PTSD), cardiovascular disease^2^ and musculoskeletal injuries.^3^ There has been a call for a critical appraisal of the health needs of females in the ADF.^4^ Military service can involve occupational and environmental exposures,^5^ including during deployment (although not everyone will be deployed and deployment may only be a small proportion of a career). Some of these exposures could impact reproductive health of either male and/or female serving personnel.^6^ Conversely, aspects of female reproductive health, for example, pregnancy, breastfeeding, could impact military readiness and/or fitness for deployment.^7^ Although clinical definitions of reproductive health vary, the definition for this review is: encompassing the reproductive and sexual health of males and females during their reproductive lives as well as fertility, pregnancy, childbirth and breastfeeding.^8^

In 2023, Joint Health Command within the ADF commissioned a catalogue of research involving serving members 2002-2023.^9^ All peer-reviewed research articles were catalogued according to at least one strategic health priority area. Among these, “gender and health” (including sexual and reproductive health of males and females and any aspects of female health) was prioritized for systematic review and evidence synthesis, which is the aim of this article.

## METHODS

Gender and sex are terms used interchangeably in casual language. Our definition was: “sex refers to the biological and physiological characteristics that define humans as male, female or intersex, based on chromosomal complement. Gender references roles, behaviour and activities that a given society, at a given time, considers appropriate for men, women and gender diverse persons.”^10^ Consequently, sex is the predominant term used.

The protocol was drafted with the Department of Defence following the PRISMA-ScR guidelines. The study protocol was registered with InPLASY (Ref: InPLASY202420077).

The detailed search strategy for the catalogue was run in: MEDLINE, Embase, PsycInfo, Cochrane Central Register of Controlled Trials, and Web of Science, April 2023. Inclusion criteria published since January 1, 2002 (pre-specified to ensure relevance to contemporary personnel); current serving (or transitioning) ADF personnel; and included a health outcome. Two authors initially screened 20% of retrieved studies at the title/abstract stage to ensure consistency. Remaining studies were screened by a single author. Three authors screened studies at the full-text stage with discrepancies resolved by consensus. Data extraction was performed by 3 authors, using a customized template (5 articles were extracted by all 3 authors to ensure consistency), and additionally, a random sample of 5% of studies was checked by an author. All included studies underwent independent quality assessment twice using Joanna Briggs Institute (JBI) risk of bias tools (https://jbi.global/critical-appraisal-tools). All retrieved studies relating to reproductive health (either sex) or female health, which included gender/sex-specific analyses were included. Methods are reported according to PRISMA-S guidelines (Preferred Reporting Items from Systematic Reviews and Meta-Analyses).

## RESULTS

**Figure 1.**
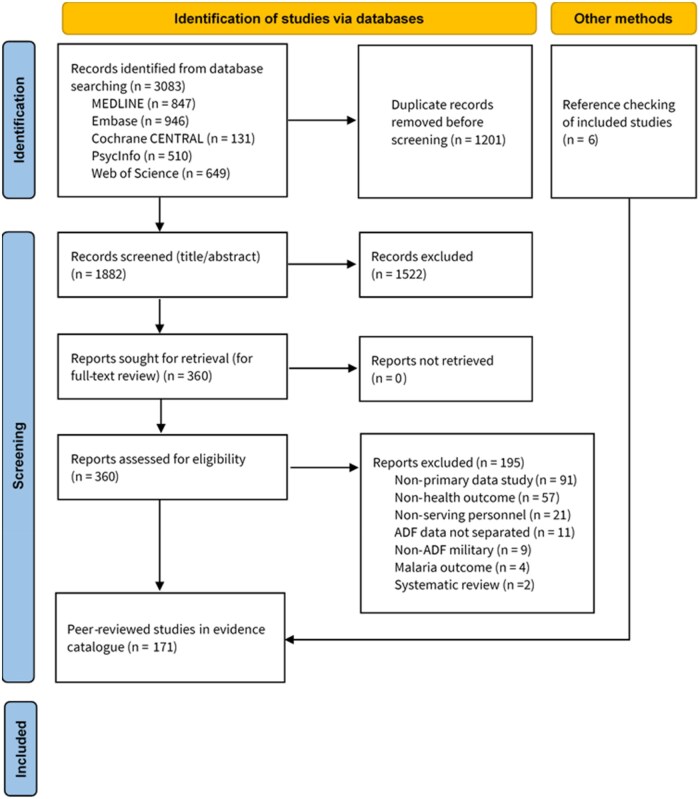
PRISMA Diagram Summarising search and yield of studies for Australian Defence Force currently-serving personnel relating to reproductive health and/or female health.

In total, 1,883 unique peer-reviewed publications were retrieved. After screening, 8 publications related to reproductive health in either sex and 14 which provided sex-stratified health data were included (**Figure 1**, **Tables 1** and **2**, **­Supplementary Tables 1 and 2**). Studies were: cohort (*n* = 9), analytical cross-sectional (*n* = 8), prevalence (*n* = 2), qualitative (*n* = 1), case-control (*n* = 1), and diagnostic test accuracy (*n* = 1). Studies without sex-stratified analyses were excluded.

**Table 1.** Description of 8 Included Studies of Reproductive Health Among Australian Defence Force Personnel 2002-2023

Citation	Study type	Participants (*n*)	Comparator	Outcome of interest
Brown et al.^11^	Cohort	1,716 male serving and ex-serving RAAF aircraft fuel tank de-seal/reseal maintenance workers	Workers who were not exposed to the hazardous chemicals	Sexual function
Bull et al.^15^	Cohort	392 ADF Servicewomen giving birth currently serving	Civilian women	Birth events
Davy et al.^16^	Analytical cross-sectional	921 currently serving ADF females deployed to the MEAO	N/A	Breastfeeding duration
Kelsall et al.^12^	Analytical cross-sectional	1,456 (I) 1,588 (C) Male Serving and Ex-serving ADF personnel deployed to Gulf War	ADF military personnel not deployed to Gulf War	Reproductive health
Stewart^17^	Prevalence	152 ADF females	General Australian population	Breastfeeding
Warner et al.^6^	Analytical cross-sectional	14,032 serving and ex-serving male and female ADF personnel deployed to MEAO	General Australian population	Fertility
Warner et al.^13^	Analytical cross-sectional	14,032 serving and ex-serving male and female ADF personnel deployed to MEAO	General Australian population	Pregnancy outcomes
Warner et al.^14^	Cohort	14,032 serving and ex-serving male and female ADF personnel deployed to the MEAO	Pre-deployment	Pregnancy outcomes

Abbreviations: ADF, Australian Defence Force; MEAO, Middle East Area Operations; RAAF, Royal Australian Air Force.

**Table 2.** Description of Included Studies of Female Health Amongst Australian Defence Force Personnel 2002-2023

Citation	Study type	Participants (*n*)	Comparator	Outcome
Burne et al.^23^	Case-control	158 male and female currently-serving cadets ADF academy	135 asymptomatic cadets	Exertional medial tibial pain
Coltman et al.^27^	Analytic cross-sectional	147 Currently serving female ADF Army soldiers	N/A	Musculoskeletal symptoms related to body armour
Coltman et al.^28^	Qualitative	33 Currently serving female ADF Army soldiers	N/A	Musculoskeletal symptoms related to body armour
Garnock et al.^24^	Cohort	123 male and female serving ADF Navy recruits	17 females and 76 males (asymptomatic)	Medial tibial stress syndrome
Newman et al.^25^	Diagnostic test	384 serving ADF Officer Army cadets (88 female) aged 17-19 before participation	ADFA asymptomatic Officer cadets (16 months follow-up)	Predicting medial tibial stress syndrome
Orr and Pope^22^	Cohort	ADF workplace injury database for all Army personnel	Male Army personnel	Relative risk of injuries
Orr et al.^21^	Cross-sectional	338 serving ADF Army soldiers exposed to large load carriage	N/A	Load carriage injuries
Orr et al. ^20^	Cohort	19,769 serving male and female ADF Army recruits mean age 22.2 ± 6 years	7,692 reserve recruits (87% male) on recruit training	Injuries, failure to pass fitness assessment
O'Shea et al.^31^	Analytic cross-sectional	491 female serving and ex-serving ADF personnel	N/A	Lower urinary tract symptoms
Schram et al.^18^	Cohort	ADF workplace injury database (SENTINEL) for all Army personnel 2018-2020	Males serving ADF Army	Injuries
Shaw et al.^26^	Cohort	107 serving male and female ADF tri-service Officer Cadets	Validation Navy officer cadets	Medial tibial stress syndrome
Tait et al.^19^	Cohort	46 serving male and female ADF Army personnel undergoing BMT	10 recruits failing to complete BMT	Failure to complete BMT
Hooff et al.^29^	Cross-sectional	24,481 serving tri-service personnel screened and 1,798 structured diagnostic mental health interview	N/A	Prevalence of 12-month and lifetime mental health disorders
Waller et al.^30^	Cross-sectional	12,829 ADF male and female personnel deployed, and non-deployed ADF contemporary military group	2010 National Drug Strategy Household Survey data	General health, role limitations, social functioning scores

Abbreviations: ADF, Australian Defence Force; ADFA, Australian Defence Force Army; BMT, basic military training.

### Quality Assessment

Overall, 10 studies were rated at low risk of bias, 10 at moderate and 2 were rated at high risk of bias because of lack of identification, or lack of consideration, of possible confounding factors (**Supplementary Table 3**).

## REPRODUCTIVE HEALTH IN MALES AND FEMALES

Reproductive health was investigated by 8 studies (**Table 1**, **Supplementary Table 1**): 3 cohort, 1 prevalence and 4 cross-sectional studies. Two studies included females only, 2 included males only, and 4 included both.

### Sexual Function

A 2009 study, assessed at moderate risk of bias, surveyed self-reported sexual dysfunction among Royal Australian Air Force (RAAF) males exposed to approximately 60 hazardous (mostly organic solvent) chemicals during de-sealing and re-sealing fuel tanks for F-111 aircraft.^11^ Among 1,692 respondents, 1,479 provided complete information for the 3 outcomes: erectile dysfunction; loss of interest in sex; problems with sexual function. After adjustment for possible confounders (hypertension, body mass index, anxiety, and depression), a 2-fold increased risk of self-reported sexual dysfunction was found in the exposed cohort compared with 2 unexposed RAAF comparison groups.^12^

### Fertility

Two studies considered fertility after deployment, one in both sexes^6^ and the other in males only.^12^ A cross-sectional study, assessed at low risk of bias, compared 1,424 male Gulf War veterans with 1,548 males from a randomly sampled ADF comparison group and found no differences in reported lifetime fertility. Males deployed to the Gulf War were 40% more likely to report affirmatively to: “trying more than 12 months to conceive” after 1991 (14% vs. 13%, OR 1.4, 95% CI, 1.0-1.8), but were also more likely to have fathered a child (OR 1.8, 95% CI, 1.2-2.6).^12^ In another study, assessed at high risk of bias, 1,730 ADF females and 12,302 ADF males previously deployed to Afghanistan and/or Iraq were asked: “have you or your partner ever had problems with infertility (attempting to conceive for more than 12 months without success)?” In total, 9% of males and 12% of females responded affirmatively (highest prevalence 18% among females aged 36-45 years). Overall, the rate of fertility problems was 9%, lower than the estimated 16% rate in the general Australian population. Sex-specific responses were only reported by age but total rates were similar after deployment to Afghanistan (7.6%) and Iraq (8.6%).^6^ However, given that the study did not control for confounders, these findings should be interpreted with caution.

### Pregnancy and Birth Outcomes

Three studies investigated the relationship between deployment, pregnancy and birth outcomes.^12^^,^^13^^,^^14^ Among male Gulf War veterans, a study assessed at low risk of bias^12^ found no differences in the pattern of live births, stillbirths or terminations experienced by the partners of those deployed as compared with partners of non-deployed males. Additionally, there were similar birth weights, gestational ages, and rates of birth defects among the veterans’ children as the comparison group’s children. In another study assessed at high risk of bias,^13^ males and females deployed to Afghanistan or Iraq self-reported a total of 15,417 pregnancies, 11,367 (74%) of which resulted in a live birth, 100 (0.8%) in a stillbirth, 69 (0.4%) in a perinatal or neonatal death and 3,077 (20%) in one of ectopic pregnancy (*n* = 273), miscarriage (*n* = 2,050), or termination (*n* = 754). Comparison with the Australian general population suggested higher risks of stillbirths (OR 3.11, 95% CI, 2.56-3.80), perinatal (OR 3.80, 95% CI, 3.26-4.44) and neonatal deaths (OR 5.43, 95% CI, 4.27-6.91) among those deployed, but lower odds of birth defects. Three major limitations of this study (confounding factors not fully measured/adjusted, lack of a military comparison group, and self-reported outcomes) prevent generalization of these findings, and suggest the results should be interpreted with caution. A follow-up study,^14^ assessed at moderate risk of bias, examined the relationship between self-reported environmental toxic exposures during deployment to the Middle East on pregnancy outcomes, analyzing as deployed to one or other conflict, or both. The study found no systematic or consistent relationship between deployment and adverse outcomes. The only adverse finding was that females deployed to both Afghanistan and Iraq reported higher rates of miscarriage than females deployed to one only but this study did not consider confounding factors or adjust for multiple testing.

A retrospective cohort study assessed at low risk of bias^15^ evaluated Queensland Health Department birth data 2012-2018 to compare birth outcomes amongst 395 females with care funded by Department of Defence (assumed to be current-­serving) and females from 3 contemporaneous comparison groups: the total state population; a propensity-scored “matched” population of mixed public/private civilians; and a private civilian sample. The researchers reported that most (97%) serving females gave birth in a private setting, had increased odds of caesarean section and epidural, and reduced odds (OR 0.57, 95% CI, 0.43-0.75) of giving birth vaginally compared to the general population. When compared with the private civilian group, rates of epidural and vaginal delivery were similar, but rates of cesarean section were higher (OR 1.45, 95% CI, 1.09-1.93) among Defence-funded deliveries, but the risk estimate had wide confidence intervals because of small numbers. The reasons for increased odds of caesarean section and epidural were not explored.

### Breastfeeding

Two studies examined self-reported breastfeeding. In a study rated at low risk of bias^16^ higher rates of breastfeeding were reported among mothers who had deployed to Iraq and Afghanistan, compared with rates in the Australian civilian population.^16^ The other study^17^ surveyed 400 ADF females who took maternity leave 2006-2007 and returned to service. Responses were received from 152 (38%), amongst whom 97% (100% of officers) reported ever breastfeeding, superior to the civilian Australian rates of 91-92%.^17^ The majority (70.7%) reported breastfeeding for >6 months, less than the >80% recommended, but better than the civilian rate (56%).^17^ Notably, 88% of ADF females returned to service within 12 months of delivery, compared with 42% among civilian females.^17^ Although assessed at low risk of bias, a major limitation of this study was the low response rate, raising concerns about participation bias which may imply the results are not representative of all ADF females.

## FEMALE HEALTH

Over 2002-2022, 14 studies reported female or sex-stratified health outcomes among ADF personnel (**Table 2**, **Supplementary Table 2**). Studied outcomes included: musculoskeletal disorders (*n* = 7); fit and function of body armour (*n* = 2); mental health (*n* = 1); risk factors for not completing basic training (*n* = 2); alcohol intake (*n* = 1); and lower urinary tract symptoms (*n* = 1).

### Musculoskeletal Disorders and Injuries

A study, assessed at low risk of bias, analysed data from an Army occupational health and safety database 2018-2020^18^ and reported similar risk of major injuries in males and females but an increased risk of minor injuries amongst females (Incidence Rate Ratio, IRR 1.53, 95% CI, 1.46-1.60). Physical training, followed by combat training, was the most frequent activities during which injuries were sustained.^18^

A retrospective cohort study, also assessed at low risk of bias, compared injury incidence in Australian Army recruits undertaking either the 80-day full-time or 28-day reserve basic training courses 2006-2011.^19^ In both courses, a higher injury incidence rate was found in females than males (1.97 vs. 1.19 injuries per person-year). Risk factors for injury were sex, age, BMI and baseline objectively measured physical fitness, but predictive models generally performed poorly. A smaller study, assessed at moderate risk of bias, examined training outcomes in 46 Australian Army recruits (9 female).^20^ Female recruits had a higher injury incidence compared with males (1.1 vs. 0.21 injuries/recruit) but adjustment for cardiorespiratory fitness mitigated any sex differences.

Two studies evaluated load carriage related injuries amongst Army personnel.^21^^,^^22^ One,^22^ rated at moderate risk of bias, identified 404 load carriage injuries occurring 2009-2010 from an administrative database, 40 of which (10%) were in females, a similar incidence to that among males. In both sexes, the commonest site of injury was the back, accounting for 27% of female injuries (*n* = 11/40), with next most common site being the foot (*n* = 8/40, 20%).^22^ However, serious personal injuries, (defined as requiring immediate treatment) were more common in females (IRR 2.40, 95% CI, 0.98-5.88). The other study, rated at high risk of bias, cross-sectionally surveyed 8 Army units, including 338 full-time soldiers (22 female), with high rates of exposure to heavy loads.^21^ At least one load carriage injury was reported by 9 females and 107 males (IRR 1.21, 95% CI, 0.71-2.24). However, this result should be interpreted with caution, given high risk of bias, small sample size and lack of accounting for confounders. No other sex-specific analyses were reported.

Four studies investigated medial tibial stress syndrome (MTSS) amongst recruits or officer cadets.^23^^,^^24^^,^^25^^,^^26^ A 12-month case-control study including 122 male and 36 female officer cadets, rated at moderate risk of bias, found that 31% females developed medial tibial pain/fractures, a risk 3-fold higher in females than males.^23^ The second study, also rated at moderate risk of bias, was a prospective cohort study involving 123 Navy recruits (28 females) undertaking an 11-week basic training course to develop a predictive risk model for MTSS and found that female sex was an independent risk factor (OR 4.4, CI, 95% 1.0-18.9).^24^ The third study, rated at low risk of bias, evaluated a shin palpation and oedema diagnostic test for MTSS in 384 Army Officer cadets (96 females) finding that both tests were more accurate among male cadets^25^ but that females had 3-fold increased risk of developing MTSS, independent of the tests. The fourth study, rated at moderate risk of bias, followed 107 asymptomatic tri-service Officer cadets, among whom 14 (41%) females and 21 (29%) males developed MTSS over 3 months.^26^ Machine learning was used to construct a prediction algorithm for MTSS, which was validated in 123 navy officer cadets and found 92% accurate (area under the curve) for predicting MTSS. Independent predictors were: female sex; years running; BMI; external rotation of the hip; orthotic prescription; number of runs/week; distance per run; and range of ankle plantar flexion.^26^

In a study rated at high risk of bias, the fit and function of body armor was investigated with 147 currently serving female soldiers.^27^ The majority of participants (96%) reported musculoskeletal pain/discomfort in the shoulder, low back and hips whilst wearing body armor. Analysis of the fit of the body armour as “too small,” “too large” versus “good fit” showed that, compared with “good fit,” “too large” was associated with greater self-reported musculoskeletal pain and abdominal discomfort (*P *= .026), hip pain (*P* < .001) and total musculoskeletal pain/discomfort (*P *= .005). “Too small” body armour was associated with more self-reported shoulder pain compared with both other groups (*P *= .016). However, equivalent data were not collected from male soldiers, making it uncertain whether these were sex-specific differences. A follow-up study,^28^ rated at moderate risk of bias, used mixed-methods to explore factors contributing to musculoskeletal pain/discomfort caused by body armour: body fit (e.g., no space for breasts) and armour shape (e.g., large size means it bounces on the body) were described as the main causes of pain/discomfort. Females in these studies were self-selected and participation bias is likely.

Amongst females deployed to Iraq/Afghanistan, there was no difference in rates of low back pain (OR 1.03, 95% CI, 0.75-1.41) or generalised myalgia (OR 1.13, 95% CI, 0.82-1.56) between females with children (*n* = 235) and females without (*n* = 686),^16^ after adjusting for service, rank and age. No male comparison data were presented.

### Mental Health and Wellbeing

A 2010 study, rated at low risk of bias, compared symptoms of PTSD, psychological distress, and alcohol misuse among 235 females deployed to Iraq and Afghanistan with dependent children and 686 females deployed without children, finding no difference in mental health symptoms.^16^

The ADF Mental Health Prevalence and Wellbeing Study surveyed half (*n* = 24,481, including 3,888 females) of all regular ADF personnel and invited a stratified, weighted sub-sample (*n* = 3,688, 438 females) to undergo a diagnostic telephone interview in a study rated at low risk of bias.^29^ Compared with males, females had an increased risk of anxiety disorders (19% vs. 14%) (OR 1.56, 95% CI 1.11-2.19), similar risk of any mental disorder (24% vs. 22%) but lower risk of an alcohol disorder (2.2% vs. 5.6%) (OR 0.36, 95% CI, 0.18-0.75).

### Alcohol Use

In a cross-sectional study, rated at low risk of bias, of alcohol use amongst 12,829 ADF personnel deployed on peace-keeping missions, both male and female personnel were less likely to be abstainers, less likely to be high-risk drinkers, and more likely to drink at lower risk levels than the general population.^30^ Females were less likely than males (OR 0.44, 95% CI, 0.30-0.64) to have harmful levels of drinking or alcohol dependence.

### Lower Urinary Tract Symptoms

A study which used a snowball sampling strategy to explore the incidence of lower urinary tract symptoms in female ADF personnel aged >18 years who had ever served (full- or part-time) for >6 months was rated at low risk of bias.^31^ Among 491 useable responses (approximately 1% of potentially eligible females), 38% were free of lower urinary tract symptoms, but 27% reported urinary incontinence, 20-27% reported bladder storage issues, and voiding impairments were reported by 9-27%, estimates comparable with those in the general female population. All symptoms were associated with age and parity. However, response bias is likely.

### Military Readiness or Fitness for Deployment

Five included studies reported about the impact of health on military readiness (**Table 3**). Two of these studies explored female-specific health outcomes (urinary incontinence and reproductive planning) but the other 3 outcomes (injuries from load carriage or recruit training and alcohol misuse), applied to males and females.

**Table 3.** Summary of Studies Including Currently-Serving Australian Defence Force Members that Considered Health in Relation to Military Readiness

Author	Health outcome(s)	Effect(s) of health outcome on military readness/deployability
Coltman et al.^27^	Ill-fitting armour	Eligibility for deployment affected by poor performance because of ill-fitting armour
Orr et al.^21^	Load carriage injuries	Injuries affect soldier and force readiness
Orr et al.^20^	Recruit training injuries	Injuries affect soldier and force readiness
O’Shea et al.^31^	Urinary incontinence	Incontinence can affect performance during deployment
Waller et al.^30^	Alcohol misuse	Alcohol misuse can limit Defence capacity
Warner et al.^6^	Reproductive ability and family planning	Deployment planning can be impacted by family planning

## DISCUSSION

Among 171 peer-reviewed papers published about currently-serving ADF members, 2002-2023, 14 presented sex-specific analyses and 8 studies explored reproductive health. Because of low numbers of studies and heterogeneity of the outcomes measured, we identified no strong evidence for any female health or reproductive health outcomes. This is not unique to Australia: similar deficits in studies of female military personnel in the United States precipitated a Delphi study setting 10-year priorities for military female health research.^32^ Notably, also, lack of separate sex-specific analysis is a major weakness of occupational epidemiological studies in all types of workers.^33^^,^^34^ Given the physical and physiological differences between males and females, in addition to differences in military representation and broader biopsychosocial considerations, we highlight a need for future studies to analyze outcomes separately by sex and, where relevant, gender.^33^

Evidence was generally conflicting as to whether deployment to wars in Iraq, Afghanistan or the Gulf War adversely impacted reproductive health. Four studies suggested no adverse effect on male fertility. Only one study, rated at high risk of bias and without a military comparison group, reported that deployment to the Middle East had increased the risk of fathering pregnancies with higher rates of stillbirth, perinatal, and neonatal death, but lower risks of birth defects than the general population.^14^ Similar results were reported in U.S. Gulf War veterans,^35^^,^^36^^,^^37^ including for birth defects.^38^ There was limited, poor-quality evidence suggesting an increased rate of miscarriage amongst females after deployment.^13^ Increased risk of adverse reproductive outcomes is biologically plausible, given that personnel may have been exposed to unknown and known hazards, possibly including nerve agents, pesticides, oil combustion, polycyclic aromatic hydrocarbons, and open pit burning.^5^ It has however, been highlighted that epidemiological and biomonitoring studies which would facilitate more insight do not yet exist.^5^

Only four studies of female reproductive health after deployment were identified: one of breastfeeding and 3 of fertility and pregnancy.^6^^,^^13^^,^^14^^,^^17^ All had methodological limitations including possible participation bias and lack of matched comparison groups. A study reported no adverse fertility outcomes after deployment to one of Iraq or Afghanistan but did not report data about females deployed to both countries.^6^ However, another study, which subsequently analysed the same data set, reported adverse effects for different reproductive health measures.^14^ A U.S. study of females deployed to Iraq and Afghanistan also reported no adverse impact on fertility, but females described urinary postponement and self-imposed fluid restriction because of limited bathroom access, which may have increased risk of vaginitis, urinary tract infections and a 22% rate of “overactive bladder.”^39^ The ADF study, which explored lower urinary tract symptoms among ever-serving females, found a similar prevalence of self-reported urinary incontinence to that among the civilian female population but had a very low response rate.^31^ In the United States, a high rate of unintended pregnancy has been reported amongst serving females compared to civilian females, with risk factors including younger age, lower socioeconomic status, lower military rank, lack of contraception use, non-white ethnicity, and intimate partner violence.^40^ It is unknown whether these findings generalize to ADF females, but additional research is needed, as numbers of female ADF members increases. Collaborative international studies could increase the power and quality of research in females.

The commonest topic of research amongst ADF females was musculoskeletal injuries, primarily among recruits/cadets. Four studies focused on MTSS during training,^23^^,^^24^^,^^25^^,^^26^ all finding a higher risk in females, consistent with that seen in the general sporting population.^41^^,^^42^ Whilst findings from these studies may be useful in reviewing training courses and/or supporting female recruits, there is a gap in research on musculoskeletal conditions, pain, and injuries whilst serving. Studies to date have focused on load carriage injuries^21^^,^^22^ and function/fit of body armor,^27^^,^^28^ with limited comparison with males to better understand and mitigate female-specific factors. The study^18^ comparing rates of injury among currently-serving males and females used data from an OHS database, which is reported to under-­represent injuries.^43^ To develop active health protection for the forces, and ensure military readiness and/or fitness for deployment, there is a need for a more strategic approach to understanding the prevalence of, and risk factors for, musculoskeletal conditions, pain and injuries. Research should consider biomechanical and psychosocial factors and include longitudinal studies. A more strategic approach could enable development of optimal prevention and treatment strategies, enhancing the health, productivity and deployability of serving personnel.

Although mental health research was prominent among ADF personnel,^9^ surprisingly few studies presented sex-stratified data. The data available suggested higher rates of anxiety, lower rates of alcohol misuse and no difference in mental health symptoms comparing females with males.^29^^,^^30^ U.S. studies found that female veterans were more likely to have experienced at least one traumatic event than civilian females (61% vs. 50% respectively),^44^ and found similar rates of PTSD as among male veterans.^45^ More sex-stratified studies with a longitudinal component are required to better understand mental health in serving females and inform preventive strategies.

Many included studies reported about health outcomes which could potentially impact military readiness and/or deployability, but female-specific health conditions (e.g., pregnancy, gynecological conditions) were more likely to be under-researched and poorly addressed.^46^ Further research investigating the relationship between female-specific health issues and military readiness is warranted.

This review must be considered alongside some limitations. The search covered the period 2002-2023 and may have missed earlier relevant studies, and only included currently- or recently-­serving personnel. Insights from studies of female veterans would be valuable. Additionally, there was a lack of high-­quality, methodologically homogeneous studies measuring the same outcomes, which prevented meta-analysis. Moreover, studies from the grey literature were excluded but may have added additional insights. However, the review benefits from a rigorous methodological approach, and to the authors’ knowledge, is novel in establishing a contemporary baseline of evidence regarding reproductive health and female health in the ADF. Our review highlights many gaps in health research involving female ADF personnel: impact of hazardous exposures; biomechanical and psychosocial effects of musculoskeletal injuries; pregnancy and gynecological health; and mental health conditions, which are highly recommended for future research, particularly as they may have implications for military readiness and/or fitness for deployment.

## CONCLUSION

This review found limited studies of reproductive and female health. Because of this, it was not possible to draw any strong conclusions about the state of female or reproductive health in the ADF. Longitudinal studies and sex-stratified analysis of all collected health data would contribute greater knowledge about female health. Gaps in the research are identified and highlighted.

## Supplementary Material

usaf411_Supplementary_Data

## Data Availability

All data generated or analyzed during this study are included in this published article.

## INSTITUTIONAL CLEARANCE

Institutional approval granted by Australian Department of Defence, March 2025.
