# Supplementary material for: Reproductive and Female Health in the Australian Defence Force 2002-2023: A Systematic Review and Synthesis
Source: Mil Med. 2025 Aug 11;191(3-4):e763–71. doi: 10.1093/milmed/usaf411 (PMC13016714; doi:10.1093/milmed/usaf411)
Supplement: usaf411_Supplementary_Data [file usaf411_supplementary_data.docx]

**Supplementary Table 1: Characteristics of 8 Studies of Reproductive Health amongst ADF personnel 2002-2023**

| Citation | Study type | Participants (n) | Inclusion/ exclusion | Intervention/ exposure | Comparator | Outcome of interest | Findings |
| --- | --- | --- | --- | --- | --- | --- | --- |
| (11) Brown et al, 2009. | Cohort | 1716 Male Royal Australian Air Force (RAAF) aircraft fuel tank de-seal/reseal maintenance workers  Serving and ex-serving | Aircraft maintenance workers  1) Exposed group: conducting fuel tank maintenance on F-11 aircraft  2) Comparison group: same workplace but not conducting fuel tank maintenance on F-11 aircraft  3) Comparison group: different workplace, similar job but no exposure | Exposure to hazardous chemicals during de-seal/re-seal fuel tank process in F-111 aircraft | Workers that were not exposed to the hazardous chemicals | Sexual function | A two-fold increase in sexual dysfunction in exposed males compared to comparison groups. (Increased risk of depression and anxiety also found in exposed males) |
| (15) Bull et al, 2022. | Cohort | 392  ADF Servicewomen giving birth  Currently serving | Females giving birth between July 2012 and June 2018.  Exposed group: ADF females giving birth  Control: civilian females giving birth in State of Queensland | Serving in the ADF | Civilian women | Birth events | ADF females have higher rates of obstetric intervention at birth: caesarean (OR 1.17, 95%CI 1.29-2.30), epidural (OR 1.56, 95%CI 1.11-2.20), lower odds vaginal birth (OR 0.57, 95%CI 0.45-0.75) and pay higher out of pocket expenses for the birth ($275.93 +/- 355.82). |
| (16) Davy et al, 2015 | Analytical cross sectional | 921 ADF females deployed to the Middle East Area Operations (MEAO), currently serving | ADF females taking maternity leave in the 2006/07 financial year. | Serving in the ADF and having a baby | N/A | Breastfeeding duration | 96.7% of participants ‘ever breastfed’ their child compared to 91-92% of general Australian population. Median breastfeeding duration was 8 months with fewer than half of the participants continued to breastfeed after returning to work (42.2%) and of these, 53.8% said they stopped because of returning to work. |
| (12) Kelsall et al, 2007 | Analytical cross sectional | 1456 (I) 1588 (C)  Male ADF personnel deployed to Gulf War  Serving and ex-serving | Exposed group: male ADF personnel deployed to the Gulf War  Control: male ADF personnel serving at same time but not deployed to Gulf War  Females excluded due to small numbers | Gulf War deployment | ADF military personnel not deployed to Gulf War | Reproductive health | Deployment to Gulf War associated with slight increased risk of fertility difficulties after deployment (OR 1.4, 95%CI 1.0-1.8) but more successful at subsequently fathering a child (OR 1.8, 95%CI 1.3-2.6). No difference in pregnancy rates and birth outcomes. |
| (17) Stewart, 2015 | Prevalence | 152 ADF females | ADF females who had taken maternity leave during the 2006/2007 financial year. | Serving in the ADF | General Australian population | Breastfeeding indicators: proportion children ever breastfed; breast fed at each month of age 0-24 months; median duration of breastfeeding | 98% breastfed for median duration of 8 months, returning to work when mean age of the child was 8.4 months. Breastfeeding prevalence (70.7%) was less than National Health and Medical Research Council target (>80%) by 6 months but compared favourably with Australian population norms on all 3 indicators until 9 months. Those who returned to work part-time had a longer median duration 10 months. |
| (6) Warner et al, 2020a | Analytical cross sectional | 14032 male and female ADF personnel deployed to MEAO  Serving and ex-serving | Participation in the Middle East Area of Operation (MEAO) census study of ADF personnel deployed to the MEAO 2001-2009. | Deployment to the MEAO | General Australian population | Fertility | Infertility rate 9% in exposed group which is lower than general Australian population (16%). |
| 13) Warner et al, 2020b | Analytical cross sectional | 14032 male and female ADF personnel deployed to MEAO  Serving and ex-serving | Participation in the Middle East Area of Operation (MEAO) census study of ADF personnel deployed to the MEAO 2001-2009. | Deployment to the MEAO | General Australian population | Pregnancy outcomes | Compared to general Australian population, adverse perinatal outcomes higher in deployed ADF personnel: stillbirth (OR 3.11, 95%CI 2.56-3.80) perinatal death (OR 3.80, 95%CI 3.26-4.44) neonatal death (OR 5.43 95%CI 4.27-6.91)  Odds of birth defects were lower for deployed ADF personnel (OR 0.52 95%CI 0.40-0.68) |
| (14) Warner et al, 2022 | Cohort | 14032 male and female ADF personnel deployed to the MEAO  Serving and ex-serving | Participation in the Middle East Area of Operation (MEAO) census study of ADF personnel deployed to the MEAO 2001-2009. | Exposure to various reproductive toxicants during deployment | Pre-deployment | Pregnancy outcomes | Self-reported adverse reproductive outcomes increased for ADF personnel deployed to Afghanistan and Iraq (p = 0.04) compared to pre-deployment, with the effect greatest for females (p = 0.009) with miscarriage being the most common (p = 0.008) |

**Supplementary Table 2: Characteristics of Studies of Female Health amongst ADF personnel 2002-2023**

| Citation | Study type | Participants (n) | Inclusion/ exclusion | Intervention/ exposure | Comparator | Outcome of interest | Findings |
| --- | --- | --- | --- | --- | --- | --- | --- |
| (23) Burne et al, 2004 | Case-control | 158 male & female cadets from ADF academy  Currently serving Cadets in ADF academy | Inclusion: Academy cadets who have passed basic physical examination and gave consent (97%).  Exclusion: Past or current history of tibial stress fracture, compartment syndrome, or significant medial tibial pain are denied entry. | Cadets in ADF academy intake who develop medial tibial pain (n=23). All had detailed tests of range of motion /foot shape etc. | Cadets in ADF academy who did not develop medial tibial pain (n=135) | Exertional medial tibial pain | Cadets followed over 12-months for predictors of medial tibial pain /fractures. Females had 3-fold higher risk. Hip range of motion predicted risk among male cadets but no other factor amongst females |
| (27) Coltman et al, 2020 | Analytical cross sectional | 147 Currently serving female ADF Army soldiers | Female ADF regular Army personnel | Wearing body armour | N/A | Musculoskeletal discomfort/pain | 96% participants reported experiencing musculoskeletal pain/discomfort, particularly shoulders, lower back, and hips – was greatest with ‘too large’ body armour fit. |
| (28) Coltman et al, 2021 | Qualitative | 147 Currently serving female ADF Army soldiers | Female ADF regular Army soldiers | Wearing body armour | N/A | Nature of problems with body armour that are associated with musculoskeletal pain/discomfort | Aspects of body armour contribute to musculoskeletal pain included: poor fit (e.g., no space for breasts), and physical shape (e.g., too large & bounces on body). Suggested multiple points of adjustment could alleviate some problems, in addition to including female anthropometrics in design. |
| (24) Garnock et al 2018 | Cohort | 123 male & female serving ADF Navy recruits | Navy personnel attending basic Navy training.  Exclusion: current medial tibial stress syndrome (MTSS), history lower limb injury or being treated for MTSS | 19 females and 13 males who developed medial tibial stress syndrome during 11-week basic training programme | 17 females and 76 males without MTSS at completion of 11-week training | Medial tibial stress syndrome | Amongst 123 recruits, 30 cases occurred and risk factors included female gender; MTSS history and increased external rotation of the hip joint. Predictive 3-item model validated that includes: MTSS history, female gender and increased hip external rotation -correctly identified 81% participants, sensitivity 82%, specificity 84%. |
| (25) Newman et al, 2012 | Diagnostic test accuracy | 384 serving ADF Officer Army cadets (88 female) aged 17-19 prior to participation | Pre-participation musculoskeletal screening by physiotherapists on ADF Army officer cadets.  Exclusion: existing injury or medical restriction | ADF Army Officer Cadets with clinical medio tibial stress syndrome (blinded diagnosis) over 16 months follow-up | ADFA Officer cadets without MTSS over 16 months of follow-up | Prediction tests for future medial tibial stress syndrome | Over 16 months there were 693 injuries in 326 cadets. 58 developed MTSS (32 males and 26 females). Positive shin palpation test was associated with a 4.63 (95% CI 2.5-8.5) higher odds of MTSS during follow-up and oedema was associated with 76.1 odds (95% CI 9.6-602.7). Both tests performed better in males. Female gender independent predictor (3 x higher risk). |
| (22) Orr & Pope, 2016 | Cohort | ADF workplace injury database for all Army personnel between 2009-2010 | Regular Army personnel, injury first experienced during, immediately after or day following a load carriage event with non indication of intervening activity. Serious personal injury (SPI), incapacity, minor injury  Exclusion criteria: Load carriage identified but injury associated with other mechanisms (e.g., running). Exposure e.g. related to exposure to workplace physical hazards, dangerous occurrence. | Female Army personnel load carriage | Male Army personnel load carriage | Relative risk of injuries | Female soldiers sustained 10% of the 401 reported injuries, with a female to male IRR of 1.02 (95% CI 0.74 to 1.41). Most common site of injury for both genders was the back, followed by the foot in female soldiers and the ankle in male soldiers. 15% (n = 6) of injuries in female soldiers and 6% (n = 23) of injuries in males were classified as SPI with the lower back the leading site for both genders. Injury risk ratio of SPI for female vs male soldiers was 2.40 (95% CI 0.98 to 5.88). |
| (21) Orr et al, 2017 | Cross sectional | 338 serving ADF Army soldiers exposed to large load carriage from 8 participating Units (artillery, armoured, engineers, infantry, signals, other, combined. 4 Units included females. | Serving fulltime in the regular Australian Army, in one of the units that is exposed to load carriage. | Exposed to load carriage | N/A | Load carriage injuries | 34% self-reported one injury during a load carriage event during their career. Relative risk among female soldiers 1.21 (95% CI 0.71-2.24). Amongst those reporting injuries, 42% reported more than one such injury. 48% of those reporting one such injury reported first injury during basic training. 61% of the self-reported load carriage injuries were to the lower limbs, 27% to the back, 9% upper limbs, 3% abdomen, and hip and 1% to the head. 55% soft tissue injuries. Field training exercise most common mechanism (28%) followed by endurance marching |
| (20) Orr et al, 2020 | Cohort | 19,769 serving male & female ADF Army recruits mean age 22.2 +/- 6 years | ADF Army or Army reserve recruit | 12,077 Army recruits receiving recruit training (91.8% males) 2006-11 over 80-days | 7,692 reserve recruits (87% male) receiving recruit training 2006-11 over 28 days | Injuries recorded in surveillance system, and failure to pass the recruit fitness assessment | Incidence of any injury 27.8% both courses. 28-day course, 17.6% ever injured vs 34.3% 80-day course). By person-years at risk, risk of any injury was higher in the 28-day course (2.29 vs 1.56 in short and long course respectively). High rates of injury among females in both courses (43.3% vs 26%). 31% of females injured in 28-day course as compared with 15.5% males in 80-day course, 55.7% of females and 32.4% of males were injured. Predictive risk factors were female gender, greater height and poorer baseline fitness. No predictive models particularly good for any outcome. |
| (31) O'Shea et al, 2023. | Analytical cross sectional | 491 female ADF personnel  Serving and ex-serving | Females over 18 years of age, having actively serving in the ADF for at least 6 months either full-time or part-time. | Serving in the ADF | N/A | Lower urinary tract symptoms | 27% reported urinary incontinence, 20-27% bladder storage issues, 9-27% voiding impairment, 41% regularly experiencing two or more symptoms, >66% lower urinary tract symptoms an ongoing issue |
| (18) Schram et al, 2022 | Cohort | ADF workplace injury database (SENTINEL) for all Army personnel between 2018-2020 | All injuries in SENTINEL database 2018-20 affecting ADF Army personnel (Total 8750 injuries)  Exclusion criteria: Record is for an incident and not for an injury | Serving in the ADF Army and being female | Serving in the ADF Army and being male. | Injuries | Rates of minor injuries were 20.75 per 100 soldiers per year in females and 13.60 per 100 soldiers per year in males: IRR for minor injuries of 1.53 (95% CI = 1.46–1.60) in female soldiers. Rates of serious injuries were 0.22 per 100 soldiers per year for female soldiers and 0.19 serious injuries per 100 soldiers per year for male soldiers (IRR 1.19, 95% CI = 0.73–1.94). Ankle was most common site of injury amongst females (14.4%) whilst knee was amongst male (12.8%). Females had higher IRRs for all mechanism of injury. Physical training was most common activity in which injuries occurred followed by combat training. |
| (26) Shaw et al, 2023 | Cohort | 107 serving male & female ADF tri-service Officer Cadets | ADF tri-service Officer Cadet Trainees, aged 18 years or above, and giving voluntary consent to participate.  Exclusion: Currently experiencing shin pain or being treated for medial tibial stress syndrome. | Volunteer tri-service officer cadets undergoing 3-months initial training | Validation study amongst navy officer cadets | Medial tibial stress syndrome (MTSS) | Data collected on all: screening for MTSS risk factors, a 4-minute physical examination (navicular drop, BMI, and passive ranges of motion for ankle plantar flexion and hip external rotation) and a 5-minute paper-based survey (past history MTSS, running experience, orthotic use). 3-month follow-up enquired about MTSS symptoms and severity. 35 incident cases over 3 months: 21 males and 14 females. Several statistical algorithms were used to develop a MTSS prediction model (based on above data) with Area Under the Curve of 0.98. |
| (19) Tait et al, 2022b | Cohort | 46 serving male & female ADF Army personnel undergoing basic military training | Australian Army recruits undertaking basic training and completing all data collection.  Exclusion: Not completing all data collection. | Completing ADF basic military training (BMT) | 10 recruits who failed to complete basic training (delayed or discharged), 6 of whom were injured | Role of subjective and objective measures in predicting failure to complete BMT within 12-weeks assessed. | 6/10 recruits who were delayed/discharged sustained injuries. Females experienced more injuries than males. Risk factors for injury during BMT included higher subjective ratings of training load, fatigue, stress, lower sleep quality, and higher cortisol concentrations. In particular, the risk of injury was twice as high in those with higher ratings of fatigue and poorer sleep quality across BMT. Higher concentrations of testosterone and higher levels of fitness upon entry to BMT were associated with reduced risk of injury. Lower baseline cardiorespiratory fitness and muscular fitness were associated with a higher risk of injury during BMT. |
| (29) Van Hooff et al, 2014 | Cross sectional | Phase 1: approximately half of all regular Navy, Army, and Air Force personnel (n=24,481)  completed self-report questionnaires.  Phase 2: stratified sub-sample (n=1,798) completed a structured  diagnostic interview (CIDI) to detect mental disorder.  (all serving personnel) | All eligible ADF personnel (N=50049) invited to participate between Apr 2010 – Jan 2011  Exclusion: ADF personnel who declined, withdrew consent, unable to be contacted. For Phase 2, also excluded if unable to be interviewed within 60 days, deployed, or started but not completed within 60 days | Survey established to make a comprehensiveassessment of the current mental health status of ADF personnel Predictors considered were sex, rank, service, and deployment status (never deployed, deployed). War-like deployment defined by specific criteria used for determinations under the Veterans Entitlement Act | N/A | Prevalence of 12-month and lifetime CIDI disorders. In this paper, 12-month CIDI diagnosed ICD-10 psychological disorder: any  affective, any anxiety, any alcohol, any mental (affective, anxiety or alcohol) disorder | Data were weighted to represent the entire ADF population (n=50,049) based on data from responders. 22% ADF members met criteria for a 12-month disorder: most commonly anxiety (14.8%), affective (9.5%) and alcohol (5.2%) disorders. At risk sub-groups were Army personnel, and those in lower ranks. Deployment status not associated with mental disorder rates. ADF females were at statistically increased odds of meeting criteria for an anxiety disorder (OR 1.56, 95% CI 1.11-2.19), but were significantly less likely to report an alcohol disorder (OR 0.36, 95% CI 0.18-0.75) compared to males. |
| (30) Waller et al, 2015 | Cross sectional | 12,829 ADF male & female personnel deployed in Bougainville or East Timor, and ADF military group who were in the ADF at the same time but not deployed Response rate for Bougainville and East Timor (41%), not stated for non-deployed group.  (50.2% full time ADF) | 12,829 individuals participating in Bougainville Deployment Health Study and East Timor Deployment Health Study invited to complete a survey on paper or online undertaken in 2008. | Hazardous and harmful alcohol consumption in previous 12 months in Alcohol Use Disorders Identification Test (AUDIT) scores: 0=abstainers, 1-7=low risk, 8-15= drink in excess of low risk, ≥16=harmful level; ≥20=probable alcohol dependence; binge drinking (≥6 drinks on one occasion). Alcohol misuse = harmful drinking, alcohol dependence, or binge drinking. | Published data from the 2010 National Drug Strategy Household Survey (NDSHS) on alcohol use: abstainers, low risk (≤2) and high risk (>2=’risky’ drinkers) standard drinks per day | General health, role limitations because of physical health problems, social functioning scores (Short Form-36 (SF-36) subscales) | Fewer abstainers, fewer risky drinkers and more people who drank at a lower risk level (≤2) in military compared with general population sample, similar in males and females. In military sample, harmful drinking and alcohol dependence more common in males, younger age, non-commissioned (NCO) officers and lower ranks, reserve and ex-serving groups. Alcohol misuse was associated with poorer general health scores, more role limitations due to physical health and lower social functioning. |

**Supplementary Table 3. Risk of bias assessment for included studies on reproductive health and female health amongst serving ADF personnel 2002-2023**

**a. Cohort studies**

| Author | Population | Exposure groups | Exposure measures | Confounders | Confounder strategies | Free of outcome | Outcome measures | Follow-up time | LTF reported | Incomplete follow up | Statistical analysis | Overall appraisal |
| --- | --- | --- | --- | --- | --- | --- | --- | --- | --- | --- | --- | --- |
| Brown et al 2009 (11) * | - | - | +/- | + | + | +/- | + | + | + | +/- | + | Mod |
| Bull et al 2022 (15)* | + | + | + | + | + | + | + | + | + | N/A | + | low |
| Garnock et al 2018 (24) | + | + | + | +/- | +/- | + | + | + | - | - | + | Mod |
| Orr & Pope 2016 (22) | + | + | + | - | - | +/- | + | + | + | N/A | + | Mod |
| Orr et al 2020 (20) | - | + | + | + | + | +/- | +/- | + | +/- | +/- | + | Mod |
| Schram et al 2022 (18) | + | + | + | - | +/- | + | + | + | + | +/- | + | Low |
| Shaw et al 2023 (26) | - | + | + | +/- | +/- | + | - | + | +/- | - | + | Mod |
| Tait et al 2022 (19) | + | + | + | +/- | +/- | + | + | + | + | N/A | + | Low |
| Warner et al 2022 (14) | N/A | N/A | + | + | - | + | + | + | + | N/A | - | Mod |

**b. Analytical cross sectional studies**

| **Author** | **Inclusion criteria** | **Description** | **Exposure measures** | **Condition measurement** | **Confounders** | **Confounder strategies** | **Outcome measures** | **Statistical analysis** | **Overall appraisal** |
| --- | --- | --- | --- | --- | --- | --- | --- | --- | --- |
| Coltman et al 2020 (27) | - | - | +/- | - | - | +/- | +/- | + | High |
| Davy et al 2015 (16)* | + | + | + | + | + | + | + | + | Low |
| Kelsall et al 2007 (12)* | + | + | + | + | + | + | + | + | Low |
| Orr et al 2017 (21) | + | + | - | - | + | +/- | - | + | High |
| O’Shea et al 2023 (31) | + | + | + | + | + | + | + | + | low |
| Waller et al 2015 (30) | + | + | + | +/- | + | + | +/- | + | Low |
| Warner et al 2020a (6)* | + | + | + | + | + | - | + | - | mod |
| Warner et al 2020b (13)* | + | + | + | + | + | - | + | - | mod |

**c. Qualitative studies**

| **Author** | **Philosophical congruity** | **Methodology & objectives congruity** | **Methodology & data collection congruity** | **Methodology & analysis congruity** | **Methodology & interpretation congruity** | **Researcher culture** | **Researcher influence addressed** | **Participant voice represented** | **Ethical research** | **Conclusion flows** | **Overall appraisal** |
| --- | --- | --- | --- | --- | --- | --- | --- | --- | --- | --- | --- |
| Coltman et al 2021 (28) | +/- | + | + | + | + | - | + | + | + | + | Mod |

**d. Prevalence studies**

| **Author** | **Sample frame** | **Participant sampling** | **Sample size** | **Subject & setting description** | **Data analysis** | **Condition identification** | **Condition measuring** | **Statistical analysis** | **Response rate** | **Overall appraisal** |
| --- | --- | --- | --- | --- | --- | --- | --- | --- | --- | --- |
| Stewart 2015 (17)* | + | + | - | + | + | + | + | + | - | Low |
| Van Hooff et al 2014 (29) | + | + | + | + | + | + | + | + | + | Low |

**e. Case Control studies**

| **Author** | **Similar groups** | **Appropriately matched** | **Same criteria** | **Exposure measures** | **exposure measure consistency** | **confounders** | **confounder strategies** | **Outcome measures** | **Exposure period** | **Statistical analysis** | **Overall appraisal** |
| --- | --- | --- | --- | --- | --- | --- | --- | --- | --- | --- | --- |
| Burne et al 2004 (23) | + | - | + | + | + | +/- | +/- | + | + | + | Mod |

**f. Diagnostic test accuracy studies**

| **Author** | **Consec/random sample** | **Case control avoided** | **Inappropriate exclusions** | **Index test interpretation** | **Threshold pre-specified** | **Correct ref standard** | **Ref standard interpretation** | **Interval between tests** | **Same ref standard** | **All patients included** | **Overall appraisal** |
| --- | --- | --- | --- | --- | --- | --- | --- | --- | --- | --- | --- |
| Newman et al, 2012 (25) | + | + | + | + | +/- | + | + | + | + | + | Low |

*Identified studies of reproductive health in ADF personnel (including males and females)
